# Supplementary material for: miR-430 microRNA Family in Fishes: Molecular Characterization and Evolution
Source: Animals (Basel). 2023 Jul 25;13(15):2399. doi: 10.3390/ani13152399 (PMC10417697; doi:10.3390/ani13152399)

Supplementary Figure S1. Variability analysis of the miR-430 sequences found in the different species of well-studied and economically relevant fishes. In the alignment the sequences are grouped base on their similarity and categorized according to how closely they resemble previously identified *Danio rerio* miR-430 variants "a" (red), "b" (blue) and "c" (yellow). Abbreviations: Aca = *Amia calva*, Aru = *Acipenser ruthenus*, Asp = *Atractosteus spatula*, Cmi = *Callorhinchus milii*¸ Dae = *Danio aesculapii*, Dal = *Danio albolineatus*, Dni = *Danio nigrofasciatus*, Dre = *Danio rerio*, Gmo = *Gadus morhua*, Gas = *Gasterosteus aculeatus*, Msa = *Micropterus salmoides*, Ola = *Oryzias latipes*, Pma = *Petromyzon marinus*, Ssa = *Salmo salar*, Str = *Salmo trutta*, Sse = *Solea senegalensis*, Tru = *Takifugu rubripes*, Tni = *Tetraodon nigroviridis*.

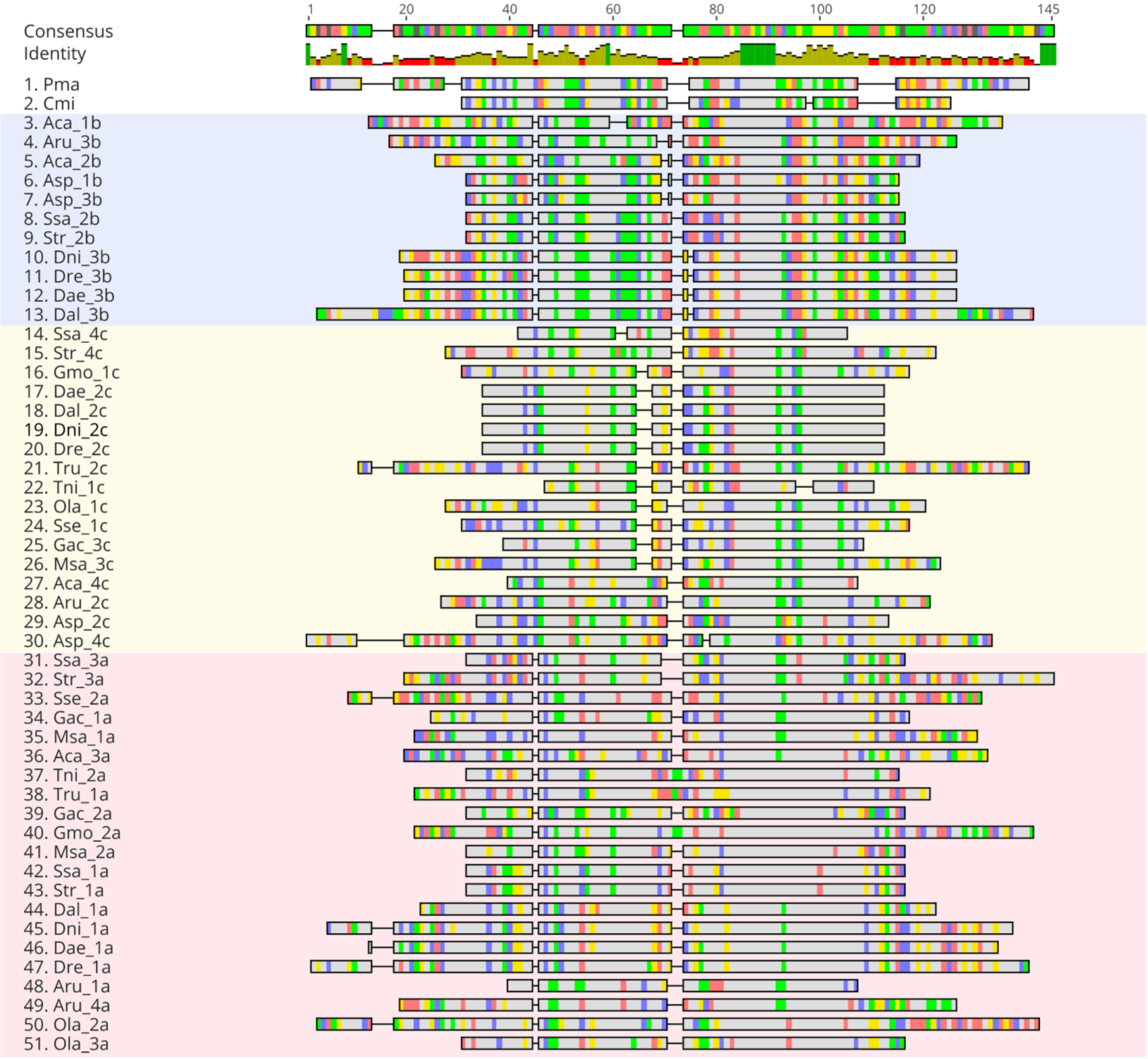

Supplement: Supplementary file 1 [file animals-13-02399-s001.zip › animals-2369801-supplementary/Figure S1.pdf]
