# Supplementary material for: miR-430 microRNA Family in Fishes: Molecular Characterization and Evolution
Source: Animals (Basel). 2023 Jul 25;13(15):2399. doi: 10.3390/ani13152399 (PMC10417697; doi:10.3390/ani13152399)

Supplementary Figure S2. Isolated repeat and repetitive elements found in *Danio rerio* chromosome 10.

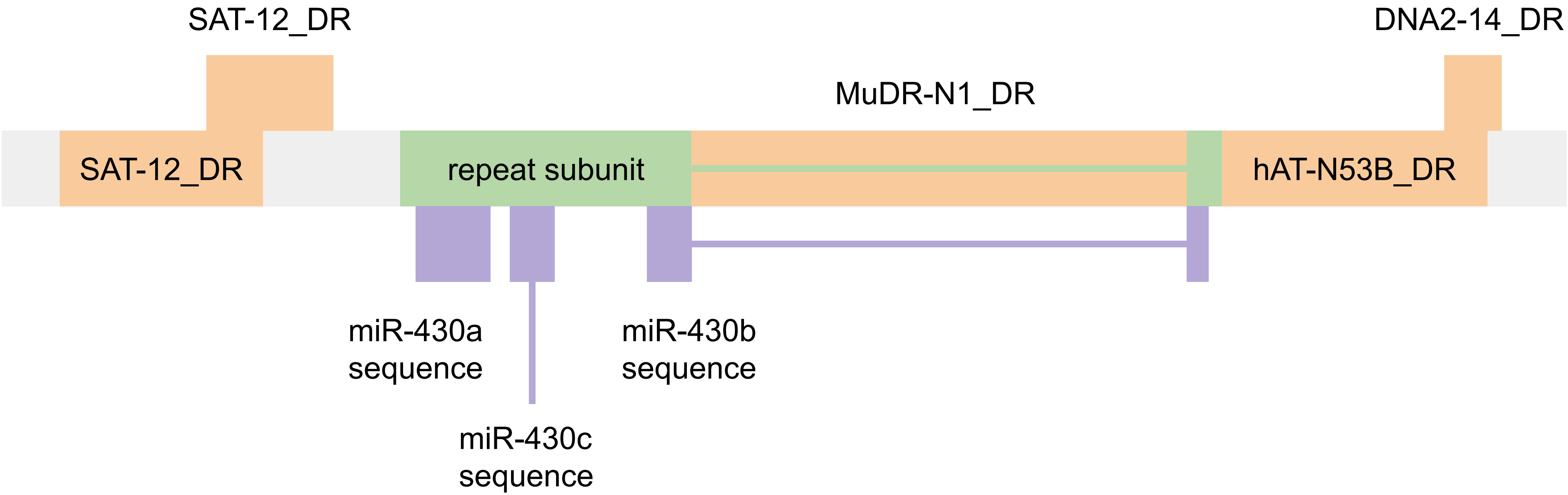

Supplement: Supplementary file 1 [file animals-13-02399-s001.zip › animals-2369801-supplementary/Figure S2.pdf]
