# Supplementary material for: miR-430 microRNA Family in Fishes: Molecular Characterization and Evolution
Source: Animals (Basel). 2023 Jul 25;13(15):2399. doi: 10.3390/ani13152399 (PMC10417697; doi:10.3390/ani13152399)
Supplement: Supplementary file 1 [file animals-13-02399-s001.zip › animals-2369801-supplementary/Table S1.pdf]

Supplementary Table S1. Data used in this study to characterized the miR-430 cluster in different fish species. Number of loci and chromosome in which the miR-430 cluster has been detected in the genome assemblies.

Species	Genome assembly	GeneBank accession number	RSA	Chromosome	Number of loci per chromosome
Acipenser ruthenus	ASM1064508v1	GCA_010645085.1	SRR8371837	2	1
Amia calva	AmiCal1	GCA_017591485.1	SRR11149502	18	1
Atractosteus spatula			SRR11149496		
Callorhinchus milii	IMCB_Cmil_1.0	GCA_018977255.1			
Danio aesculapii	fDanAes4.1	GCA_903798145.1	ERR9973238	4	1
				10	1
Danio albolineatus			ERR027129		
Danio nigrofasciatus			ERR036175		
Danio rerio	GRCz11	GCA_000002035.4	ERR012983	4	1
				10	1
Gadus morhua	gadMor3.0	GCA_902167405.1	SRR13374370	17	1
Gasterosteus aculeatus	GAculeatus_UGA_version5	GCA_016920845.1	SRR19737622	4	1
Micropterus salmoides			SRR20217406		
Oryzias latipes	ASM223467v1	GCA_002234675.1	SRX197403	4	2
Petromyzon marinus	kPetMar1.pri	GCA_010993605.1	SRR6329400	8	1
Salmo salar	Ssal_v3.1	GCA_905237065.2	ERR8684086	3	1
				6	1
				19	1
				21	1
Salmo trutta	fSalTru1.1	GCF_901001165.1	ERR7645480	1	1
				32	1
Takifugu rubripes	fTakRub1.2	GCF_901000725.2	SRR8585992	11	1
Tetraodon nigroviridis			SRR16668014		

Data used in cichlids species.

Species	Genome assembly	GeneBank accession number	RSA	Chromosome	Number of loci per chromosome
Amphilophus citrinellus	ASM1343575v1	GCA_013435755.1	ERR4189679	LG13	1
Astatotilapia stappersii			SRR9675392		
Ctenochromis horei			SRR9657492		
Cyprichromis leptosoma			SRR9665717		
Haplochromis burtoni			SRR9674053		
Labeotropheus trewavasae			SRR17068914		
Melanochromis auratus			SRR17068925		
Oreochromis korogwe			ERR4508029		
Oreochromis niloticus	O_niloticus_UMD_NMBU	GCA_001858045.3	SRR071588	LG13	2
Perissodus microlepis			SRR9665675		
Pseudocrenilabrus philander			SRR7185089		
Pundamilia nyererei			SRR4169625		
